# Comprehensive Clinical Characterization and Long-Term Follow-Up of the Institut Català d’Oncologia Breast Cancer Observational Cohort Study

**DOI:** 10.3390/cancers17081366

**Published:** 2025-04-19

**Authors:** Helena Pla, Bartomeu Fullana, Anna Esteve, Roser Fort-Culillas, Angelica Ferrando-Díez, Adela Fernández-Ortega, Anna Pous, Agostina Stradella, Rafael Villanueva-Vázquez, Beatriz Cirauqui, Catalina Falo, Evelyn Martínez-Pérez, Guadalupe Molina, Sonia del Barco, Arantxa Eraso, Mireia Margelí, Gemma Viñas, Miguel Gil-Gil, Lourdes Petriz, Sonia Pernas

**Affiliations:** 1 Breast Cancer Unit, Department of Medical Oncology, Institut Català d’Oncologia, 17007 Girona, Spain; hpla@iconcologia.net (H.P.); rfort@iconcologia.net (R.F.-C.); sdelbarco@iconcologia.net (S.d.B.); gvinyes@iconcologia.net (G.V.); 2 Precision Oncology Group (OncoGIR-Pro), 17007 Girona, Spain; 3 Biomedical Research Institute (IDIBGI-CERCA), 17190 Salt, Spain; 4 Breast Cancer Unit, Department of Medical Oncology, Institut Català d’Oncologia, L’Hospitalet de Llobregat, 08908 Barcelona, Spain; afernandezortega@iconcologia.net (A.F.-O.); astradella@iconcologia.net (A.S.); ravillanueva@iconcologia.net (R.V.-V.); cfalo@iconcologia.net (C.F.); mgilgil@iconcologia.net (M.G.-G.); 5 Institut d’Investigació Biomèdica de Bellvitge (IDIBELL), L’Hospitalet de Llobregat, 08908 Barcelona, Spain; 6 Badalona Applied Research Group in Oncology (B-ARGO), Translational Program in Cancer Research (CARE), Germans Trias i Pujol Research Institute (IGTP), 08916 Badalona, Spain; aesteve@iconcologia.net; 7 Breast Cancer Unit, Department of Medical Oncology, Institut Català d’Oncologia, 08916 Badalona, Spain; aferrandod@iconcologia.net (A.F.-D.); apousb@iconcologia.net (A.P.); bcirauqui@iconcologia.net (B.C.); mmargeli@iconcologia.net (M.M.); 8 Breast Cancer Unit, Department of Radiation Oncology, Institut Català d’Oncologia, L’Hospitalet de Llobregat, 08908 Barcelona, Spain; emperez@iconcologia.net; 9 Breast Cancer Unit, Department of Radiation Oncology, Institut Català d’Oncologia, 08916 Badalona, Spain; jgmolinao@iconcologia.net; 10 Breast Cancer Unit, Department of Radiation Oncology, Institut Català d’Oncologia, 17007 Girona, Spain; aeraso@iconcologia.net; 11 Oncology Results Office, Directorate of Clinical Strategy, Research and Clinical Results, Institut Català d’Oncologia, L’Hospitalet de Llobregat, 08908 Barcelona, Spain; lpetriz@iconcologia.net

**Keywords:** long-term follow-up, breast cancer, survival outcomes, real world data, observational study

## Abstract

**Background/Objectives**: Few large cohorts with relatively uniform treatment approaches and long-term follow-up are available for assessing clinical outcomes for breast cancer (BC) patients. The Institut Català d’Oncologia (ICO) Breast Cancer Cohort was designed to well characterize treatment patterns and overall survival outcomes at 5 and 10 years, with a particular focus on patients < 40 and ≥70 years old, age groups often underrepresented in clinical trials. **Methods**: In this retrospective, observational study, we included all pathologically confirmed invasive BC patients diagnosed and treated between 2010 and 2014 at ICO, a Spanish reference cancer center, with a follow-up until November 2023. We collected comprehensive real-world data on clinicopathologic characteristics and treatment modalities. Overall survival (OS) was estimated using the Kaplan–Meier technique and was reported stratified by prognostic factors for the age groups of ≤40, 41–69 and ≥70. The Multivariate Cox model was used to estimate the risk of death for subgroups of age, adjusting for subtype, stage and grade. **Results**: Overall, 3451 patients with stage I to IV BC were diagnosed and treated, with a mean age of 58 years (range 19–98); 371 (10.8%) were diagnosed ≤40 years, and 756 (21.9%) were ≥70 years. With a mean follow-up of 9.9 years (SD = 3.5), the 5- and 10-year OS were 89% (95% CI: 86–92%) and 85% (95% CI: 81–88%) for patients ≤ 40, respectively; for those aged 41–69 years, 91% (95% CI: 90–92%) and 85% (95% CI: 83–86%), respectively; and 70% (95% CI: 66–73%) and 50% (95% CI: 47–54%) for those ≥70 years, respectively. The 5- and 10-year relative survival (RS) were 92% and 88% for patients < 70 years, respectively, and 82% and 77% for those ≥70 years, respectively. The Multivariate Cox model identified a HR of 4.90 (95% CI: 3.44–6.97, *p* < 0.001) for patients ≥ 70 years compared to those between 41 and 69 years. **Conclusions**: The ICO Breast Cancer Cohort, as far as we know, the largest in Spain with long-term follow-up, underscores the critical role of age and subtype in determining overall survival outcomes in patients with breast cancer.

## Simple Summary

This study examines treatment patterns and overall survival outcomes in breast cancer patients, focusing on age groups often underrepresented in clinical trials—those under 40 and over 70 years old. By analyzing data from over 3400 patients, it explores how treatment approaches and survival vary according to age and stage at diagnosis. This study provides valuable real-world evidence on long-term outcomes at 5 and 10 years, in these age extremes. The findings offer insights that may help refine treatment strategies and improve patient care, particularly for those in underrepresented age groups.

## 1. Introduction

Given the biologic heterogeneity within patients with breast cancer, the era of personalized medicine aims to enhance the prediction of clinical results and guide individualized treatment. In addition, the significance of follow-up data becomes especially evident, as it is fundamental in comprehending these differences. In contrast to the numerous prospective cohorts dedicated to cancer etiology, there are few large retrospective cohorts for assessing clinical outcomes with an extended follow-up [1,2,3]. In this heterogeneity, the age factor is usually underestimated, but it is worth noting that worst breast cancer (BC) outcomes tend to cluster at the age extremes. Alongside younger patients, elderly patients also experience less favorable outcomes [4,5].

The impact of BC on young women (defined as <40 years) is particularly alarming, standing as the leading cause of cancer-related deaths among this population. Two factors exacerbate this situation: young women often lack screening and early detection, leading them to present with higher-stage and symptomatic BC. Emerging data suggest that the impact of age might vary depending on the tumor subtype. Various population-based studies indicate that younger women tend to develop more aggressive BC subtypes, including triple-negative (TNBC) and HER2-positive (HER2+) variants [6,7]. Moreover, within hormone receptor-positive (HR+) cancers, these women often exhibit tumors of a higher grade, and young women under the age of 40 with HR+ cancers face a risk of death that is nearly 1.5 to 2 times greater than women over 40 [4,5,8,9,10].

On the other hand, with the aging of our population, we expect a rise in older patients (defined as >70 years) diagnosed with BC [9]. Interestingly, while age seems to be inversely related to the occurrence of more aggressive BC types, the outcomes for elderly patients remain inconsistent. Those diagnosed with local advanced or metastatic forms face even worst consequences, and older age is associated with early mortality in those presenting with metastatic disease. Furthermore, many elderly patients, regardless of the disease stage, receive suboptimal or non-standard treatment. This is often due to concerns about existing health issues, potential treatment side effects and fragility [4,11,12,13].

Given this spectrum of varied outcomes across subgroups and aware of the absence of extended follow-up cohorts, our institution, the Institut Català d’Oncologia (ICO), developed an extensive patient cohort dedicated to BC. ICO is a nationally recognized public monographic cancer institute that adopts a comprehensive approach, encompassing prevention, diagnosis, treatment and follow-up, specialized training and research (epidemiological, clinical and translational). ICO comprises three centers collaborating closely with three hospitals: Hospital Universitari de Bellvitge (HUB) in L’Hospitalet, Barcelona; Hospital Universitari Doctor Josep Trueta (HUDJT) in Girona; and Hospital Universitari Germans Trias i Pujol (HUGTiP) in Badalona. Furthermore, it operates within a network involving 20 regional hospitals, providing care to approximately 3 million people in Catalonia, Spain. The present study aimed to systematically gather real-world clinical data with long-term follow-up from patients with BC treated at ICO between 2010 and 2014, to generate evidence-based scientific knowledge.

Considering the clinical heterogeneity observed in breast cancer outcomes, this study hypothesizes that patients at the age extremes (<40 years and ≥70 years) may present poorer clinical outcomes. This study aims to assess whether patients at the age extremes with breast cancer experience worse clinical outcomes in terms of overall survival (OS), in a large cohort with long-term follow-up. This research will also investigate how age interacts with tumor subtype, stage and histological grade.

## 2. Materials and Methods

### 2.1. Eligible Patients and Data Collection

This multicenter cohort study was commissioned by the ICO institution to estimate and provide our OS rates as indicators of clinical outcomes and quality, reported following ESMO Guidance for Reporting Oncology Real-World evidence (GROW) [14]. The inclusion period was from 1 January 2010 to 31 December 2014 and involved patients who were attended at one of the 3 ICO centers and followed-up data until 30 November 2023. Eligible patients were female ≥ 18 years old; diagnosed with invasive BC (any subtype), with available information on stage at diagnosis; and who received treatment at any of the ICO centers or a reference hospital center (including surgeries and systemic treatments at the regional network centers associated with the ICO).

Sources of information to identify eligible patients comprised (i) pathology reports, (ii) hospital discharges and (iii) Registry of Tumors of ICO Girona. Demographic and clinical information was obtained by reviewing medical records from the following sources: (i) Electronical Medical records at ICO centers; (ii) Shared Clinical History in Catalonia; (iii) Chemotherapy prescriptions information system (ESPOQ); and (iv) Radiotherapy prescription software (ARIA). The Unit of Information Systems at ICO designed, implemented and maintained a specific database (DB) for the purposes of the study. Data were manually entered by expert documentalists under medical supervision into the DB.

Demographic and clinical data were collected, including baseline diagnostic and prognostic factors; initial treatment: surgery, systemic treatments (chemotherapy, hormone therapy or targeted therapies), radiotherapy (external or brachytherapy); treatment responses; vital status; and the date of last follow-up (Table S1). All variables regarding treatments performed were collected from records of the reference hospital or from any of the external centers. The date of diagnosis was defined as the date of the first positive biopsy. The Unit of Information Systems at ICO routinely update the date of death through systematic linkage with the National Death Index (INDEF). For patients with no evidence of date of death, the date of last follow-up was updated from the date of last control at ICO and the date of last control at a non-ICO center, whichever occurred last. For the present analysis, the date of administrative censoring was 30 November 2023. The Research Ethics Committee of the Hospital Universitari de Bellvitge reviewed and approved all the documentation related to this study (Ref PR108/24).

### 2.2. Statistical Analysis

The description of the population characteristics was carried out using frequencies and percentages for categorical variables, and medians with their ranges and interquartile ranges (IQRs) for continuous variables. The survival time was defined as the time in years from the date of tumor diagnosis to the date of death or the date of administrative censorship (30 November 2023), whichever occurred first. The OS was estimated using the Kaplan–Meier statistic. Median OS and their 95% confidence intervals (CIs) were reported.

Net survival was estimated through the relative survival (RS) approach, incorporating expected rates from the general population matched by age, sex and calendar year. The Pohar–Perme method and the 2023-updated Spanish life tables from the Human Mortality Database were used [15]. RS at 2, 5 and 10 years were reported. The Multivariate Cox proportional hazards model was used to estimate the risk of death for subgroups of age, adjusting for subtype, stage and histologic grade at cancer diagnosis. Interaction terms between age and the potential confounders were included in the model and retained if the likelihood ratio test was statistically significant.

## 3. Results

Out of 4065 patients identified during the study period, 3451 met the inclusion criteria (Figure S1). These 3451 patients had a mean age of 58 years [range 19–98]. Of them, 371 (10.8%) were diagnosed at or before 40 years old, 2324 (67.3%) between 41 and 69 years old and 756 (21.9%) at 70 years or older. Regarding BC subtypes, 2358 (68.3%) were HR+/HER2−, 459 (13.3%) were HER2+ and 403 (11.6%) TNBC, with 231 (6.6%) unclassified. Most patients were diagnosed at stage I (1111 patients, 32.2%) and stage II (1593, 46.2%), while stage III (583, 16.9%) and stage IV (164 patients, 4.8%) were less frequent. With a mean follow-up of 9.9 years (SD = 3.5), the median OS was not reached; the 5-year OS was 89% (95% CI: 86–92%).

### 3.1. Clinicopathological Characteristics by Age Group

A total of 371 patients (10.8%) were diagnosed with breast cancer at or before 40 years old. Within this younger cohort, the mean age was 37 years [IQR 33.0–39.0]. The most common invasive BC subtype was HR+/HER2− in 221 patients (59.6%), followed by TNBC in 73 patients (19.7%) and HER2+ in 61 patients (16.4%). The majority of younger patients were diagnosed at stage II (58%) and stage III (22.4%). Invasive ductal carcinoma was the most frequent histological subtype (88.9%), while only 2.2% of patients had invasive lobular carcinoma. Grade III was the most common histological grade (44.7%), and the Ki67 score was ≥20% in 69.8% of cases (Table 1). Regarding initial treatment, 53.9% of patients underwent upfront surgery, whereas 42.9% received neoadjuvant chemotherapy. Breast-conserving surgery (BCS) was the most commonly performed surgical technique (51.9%), and 59.5% of patients underwent axillary dissection. (Table 2).

In the subgroup of patients aged 41 to 69 years (2324, 67.3%), the mean age was 55 years [IQR 48.0–62.0]. The HR+/HER2− subtype was also the most frequently diagnosed BC subtype (69.1%), followed by the HER2+ subtype (13.8%) and TNBC (10.5%). Most patients in this age group were diagnosed at stage II (44.3%) and stage I (36.1%). Invasive ductal carcinoma was the most common histological subtype (81.5%), with histological grade II in 41.1% of patients and grade I in 21.1%. A Ki67 score ≥ 20% was noted in 46.9% of tumors (Table 1). Surgery was the upfront treatment in 75.7% of patients, followed by neoadjuvant treatment in 21%. BCS was the most used surgical approach (68.2%), with 41.6% of patients undergoing axillary dissection (Table 2).

Among older patients, 756 (21.9%) were diagnosed at age 70 or older. In this subgroup, the mean age was 77 years old [IQR 73.0–81.0]. The most frequently diagnosed subtype was HR+/HER2− (70.4%), followed by TNBC (11.4%) and HER2+ (10.2%). These patients were mainly diagnosed at stage II (46%) and stage I (28.2%), with 8.5% presenting at de novo stage IV. Invasive ductal carcinoma was the most common histological subtype (71.6%), with histological grade II (37.3%) and grade I (20.1%) being the most prevalent, and a Ki67 score ≥ 20% was seen in 42.3% of cases (Table 1). Surgery was the first treatment in 72.1% of cases, with neoadjuvant treatment used in 19.3%. BCS was the preferred approach (60.2%), and axillary dissection was performed in 39.2% of patients (Table 2).

### 3.2. Clinicopathological Characteristics by Breast Cancer Subtype (Table S2)

In the subgroup of HR+/HER2− tumors, the mean age was 58 years [range 19–98], while it was 54 years [range 22–95] in the HER2+ group and 56 years [range 27–97] in the TNBC group. Across all subtypes, the majority of patients were diagnosed at stages I and II, although stages III and IV were more frequent in the HER2+ and TNBC subgroups.

Regarding histology, most patients (80.1%) had invasive ductal carcinoma, while 9.2% had invasive lobular carcinoma. Histological grade I was more common in the HR+/HER2− subtype (25.9%) compared to HER2+ and TNBC (3.9% and 3.5%, respectively). Notably, 70.7% of TNBC had grade III tumors, and 75.2% had Ki67 levels ≥ 20%.

In terms of surgical approach, BCS was performed in 66.6% of HR+/HER2− patients, 65.5% of TNBC and 56.7% of HER2+ patients, whereas mastectomy was used in 29.6% of HER2+, 19.5% of HR+/HER2− and 19.2% of TNBC patients. Sentinel lymph node biopsy (SLNB) was the most frequent approach for axillary staging across all BC subtypes. Lymphadenectomy was performed in 56.8% of HER2+ patients, 49.3% of TNBC and 39.7% of HR+/HER2− patients. Upfront surgery was the first treatment in 79.3% of HR+/HER2− cases, 54.2% in HER2+ cases and 51.9% of TNBC. Neoadjuvant treatment was used in 42.2% of TNBC, followed by HER2+ (39.9%) and HR+/HER2− patients (17.3%).

### 3.3. Clinicopathological Characteristics by Stage (Table S3)

Regarding stage, the mean age at diagnosis was similar across stages: 59 years in stage I [range 27–93], 56 years in stage II [range 19–97], 56 years in stage III [range 25–96] and 63 years in stage IV [range 32–98].

With respect to surgical approach, lumpectomy was performed in 77.4% of stage I patients and 66.1% of stage II, whereas nearly half of stage III patients (47.4%) underwent mastectomy. Among stage IV cases (164 patients), 31 patients underwent mastectomy and 17 lumpectomy, accounting for 29.2% of that group. In terms of SLNB, it was performed in 90.4% of stage I and 69.3% of stage II cases, decreasing to 23.9% in stage III. Notably, axillary lymph node dissection was most frequently performed in stage III patients (93.2%), decreasing to 5.4% in stage I.

### 3.4. Overall Survival and Prognostic Factors

**Figure 1:**
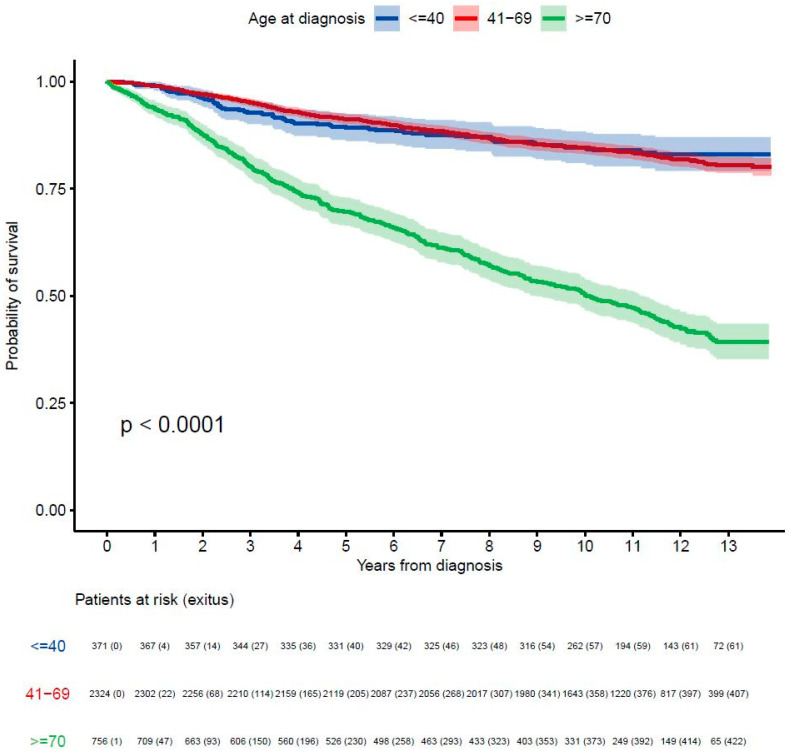
Probability of survival according to groups of age.

Survival analysis by age showed the following OS rates: for patients ≤ 40 years, 89% (95% CI: 86–92%) at 5 years and 85% (95% CI: 81–88%) at 10 years; for those 41–69 years, 91% (95% CI: 90–92%) at 5 years and 85% (95% CI: 83–86%) at 10 years; and for patients ≥ 70 years, 70% (95% CI: 66–73%) at 5 years and 50% (95% CI: 47–54%) at 10 years (Figure 1).

**Figure 2:**
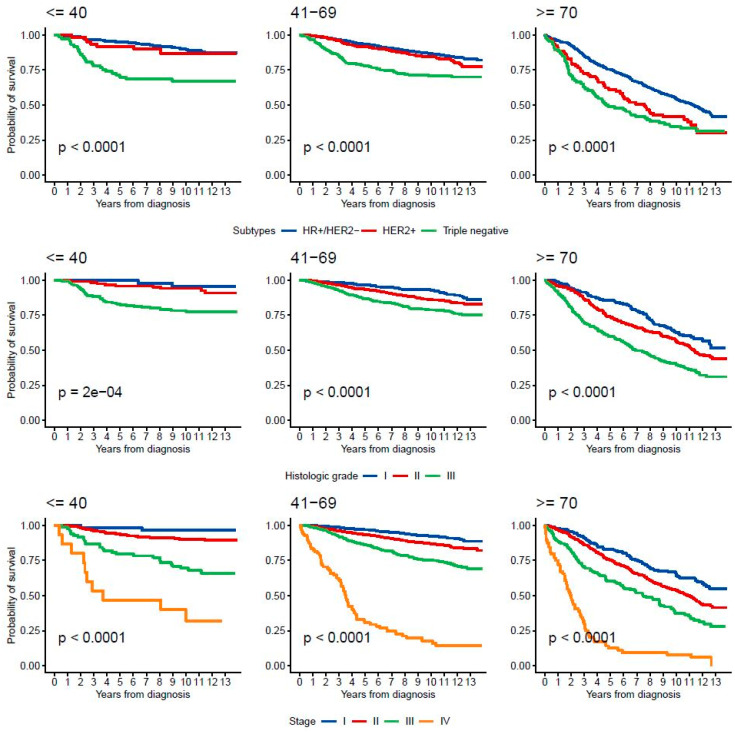
Survival probability by age group, histologic grade and stage.

In the ≤40 years age group, the median OS was not reached for stages I to III, but it was 3.7 years (95% CI: 2.3–NR) for stage IV (Table S4). For the 41–69 years group, the median OS was not reached for stages I to III and was 3.5 years (95% CI: 3.1–4.2) for stage IV. In patients ≥ 70 years, the median OS was 11 years for stage II, 7.5 years for stage III and 2 years for stage IV. Only the ≥70 years group achieved a statistically significant median OS of 10 years (95% CI: 8.9–11), compared to younger groups, which had not yet reached the median OS (*p* < 0.0001). OS at 5 and 10 years by stage are detailed in Table 3, Figure 2 and Figure S2.

The 5- and 10-year OS for the HR+/HER2− subgroup were 94% and 82% in stage I, 92% and 72% in stage II, 84% and 62% in stage III, and 29% and 15% in stage IV, respectively. For the HER2+ subgroup, OS at 5 and 10 years were 95% and 86% in stage I, 91% and 76% in stage II, 78% and 60% in stage III, and 41% and 28% in stage IV, respectively. In the TNBC subtype, OS at 5 and 10 years were 92% and 73% for stage I, 72% and 58% for stage II, 64% and 41% for stage III, and 4.7% at both 5 and 10 years in stage IV, respectively (Table S5).

Additionally, our analysis of tumor grade—categorized as low (grades I and II) and high (grade III)—on OS in HR+/HER2-BC patients, stratified by age, revealed significant differences in OS within the low-grade group. Specifically, patients younger than 40 had better outcomes than those aged 41–69 (Figure S3).

### 3.5. Relative Survival (RS) and Risk Factors of Death

In the absence of cancer-specific mortality information, we estimate the RS by taking into account expected mortality rates from the general population to account for mortality from causes other than cancer. The RS at 5 and 10 years were 92% (95% CI: 91–93%) and 88% (95% CI: 86–89%) for patients < 70 years, and 82% (95% CI: 78–87%) and 77% (95% CI: 69–86%) for those ≥70 years, respectively (Figure S4).

The results of the multivariate Cox model identified age, subtype, stage and histology as risk factors for death (Table 4). The HR for women with ≤40 years was 0.29 (95% CI: 0.07–1.25, *p* = 0.0968) and for ≥70 years was 4.90 (95% CI: 3.44–6.97 *p* < 0.001), compared to the reference group of 41–69 years. The HR for TNBC was 1.63 (95% CI: 1.20–2.22, *p* = 0.0018), indicating a significantly increased risk of death compared to the HR+/HER2− subtype. Stage and histology showed a gradient association with an increasing risk of death. The interaction between age ≤ 40 and the triple-negative subtype yielded an HR of 2.25 (95% CI: 1.11–4.57, *p* = 0.0240). The interaction between age ≥ 70 and stage IV resulted in an HR of 0.36 (95% CI: 0.20–0.63, *p*-value = 0.001).

## 4. Discussion

Our study highlights significant age-related differences in BC outcomes between younger (≤40 years) and older (≥70 years) patients. Age-specific analysis revealed that younger patients presented with more aggressive disease features, including a higher histological grade (with almost 45% having grade III tumors) and higher Ki67 levels (69.8% with a Ki67 ≥ 20%). Additionally, the frequency of the TNBC (19.7%) and HER2+ (16.4%) subtypes was higher among younger patients, consistent with findings from other studies [6,7]. This aggressive disease presentation is further supported by the fact that only 5.2% and 13.5% of patients ≤ 40 years were diagnosed at stage I and II, respectively, suggesting both a potential delay in diagnosis and a biologically more aggressive disease course in this population.

Across all breast cancer subtypes, 43% of younger patients received neoadjuvant treatment, in contrast to only 19.3% of patients aged ≥70 years. Despite the aggressive nature of their cancer, younger patients showed high survival rates, with a 5- and 10-year OS rate for those diagnosed at stages I to III not reaching the median survival. Contrary to some published studies, our cohort of younger patients did not exhibit a worse prognosis [6,16]. We hypothesize that factors such as universal access to healthcare and equitable BC treatment protocols at our institution may contribute to these favorable outcomes. However, in our study, younger patients with triple-negative breast cancer have a particularly high risk of death, aligning with findings from previous studies [17,18].

In contrast, patients aged ≥70 years—who comprised 22% of our cohort—experienced poorer outcomes, with 5- and 10-year OS rates of 70% (95% CI: 66–73%) and 50% (95% CI: 47–54%), respectively. Although these differences were smaller, they remained statistically significant, even after accounting for non-cancer mortality using the relative survival (RS) approach. Moreover, in our study, older patients had a statistically significant higher risk of death. These findings agree with previously reported data indicating a worse prognosis for patients ≥ 70 years, although discrepancies in the literature regarding outcomes in elderly patients still exist [19,20,21,22]. Older patients are more likely to die of non-BC causes, particularly when coexisting with other comorbidities [23,24]. These findings highlight the need for strategies that address comorbidities and overall health improvement in elderly BC patients. An oncogeriatric assessment and a multidisciplinary approach are crucial in optimizing treatment for elderly breast cancer patients, as they allow for a comprehensive evaluation of comorbidities, functional status and individual patient needs, ultimately improving clinical outcomes and enhancing quality of life [21,25,26,27,28,29]. Notably, elderly patients were less treated with chemotherapy, while the administration of Trastuzumab remained consistent across age groups (Table 2). Compared to a Dutch population-based study in elderly patients, in our cohort, a higher percentage received chemotherapy, and nearly all stage I–III cases underwent surgery, as opposed to only 66.0% in the mentioned cohort [30].

Our findings emphasize the heterogeneity of BC, particularly in terms of subtype-specific outcomes. Patients with HR+/HER2− tumors exhibited the most favorable survival outcomes, whereas those with TNBC had significantly poorer prognoses. HER2+ patients benefited from the advent of anti-HER2-targeted therapies, with a median OS similar to that of HR+/HER2− patients, demonstrating the transformative impact of these treatments on HER2+ BC management. The potential impact of current novel therapeutic strategies for TNBC may become evident in the coming years though comprehensive clinical cohort studies.

As expected, stage at diagnosis played a critical role in survival outcomes, with the majority of patients diagnosed at early stages (I and II), likely contributing to the overall favorable OS rates observed in our cohort. Yet, survival rates for stage IV patients remained low across all subtypes, indicating the persistent challenges in managing metastatic disease and underscoring the need for continued innovation in systemic treatment approaches. Treatment patterns in our cohort reflected a preference for BCS in early-stage disease, along with increased use of neoadjuvant therapies in more advanced stages or in aggressive subtypes like TNBC and HER2+.

Comparing our cohort with other BC registries, such as the American National Cancer Database (NCBD) [1] and the Japanese National Clinical Database (NCD) [3], we observed that the mean age at diagnosis was comparable (58 years in our cohort versus 61 and 65 years in NCBD and NCD, respectively). Regarding stage, the rate of stage IV diagnoses (4.8%) in our cohort was also similar to the American cohort (4%) but higher than the Japanese cohort (2.1%). In all three cohorts, most patients were diagnosed at stages I and II, although subgroup analyses by age were not reported. Regarding OS in HR+/HER2− patients, we observed significant differences between younger patients (<40 years) and those aged 41–69 within the low-grade subgroup, with younger patients showing better outcomes. Interestingly, research by Partridge et al. [6] reported a higher risk of BC-related death among younger patients, particularly in cases involving low-grade HR+/HER2− tumors. This discrepancy warrants further investigation into potential factors influencing survival outcomes differently in our cohort compared to others.

The strengths of our study include a long follow-up period exceeding 10 years, a large patient population and comprehensive epidemiological and clinical data collection within a multicenter single-institution framework that applies uniform diagnostic and treatment protocols. To our knowledge, this is the largest BC cohort study conducted to date in Spain. However, the study is not without limitations, including unavailable data on causes of death, genetic counseling, clinical trial participation and lifestyle factors.

Advancements in treatment strategies, such as the introduction of cyclin-dependent kinase inhibitors, antibody drug conjugates, immunotherapy and novel anti-HER2 therapies, are likely to impact patient outcomes and survival trends. Updating the ICO Breast Cancer Cohort would therefore be valuable to capture more detailed and contemporary data reflective of these therapeutic innovations.

## 5. Conclusions

This real-world cohort provides valuable insights into the epidemiology, clinicopathological characteristics, treatment patterns, overall survival and long-term follow-up of BC patients, with particular focus on the underrepresented age groups of <40 and ≥70 years. While younger patients did not exhibit inferior survival outcomes—except in TNBC cases—elderly patients experienced poorer survival rates, largely influenced by comorbidities. The ICO Breast Cancer Cohort, conducted within a multicenter single-institution framework with standardized protocols, offers relevant data that may support future research and contribute to a better understanding of age-related disparities in breast cancer.

## Figures and Tables

**Table 1:** Baseline clinicopathological characteristics by age subgroup.

	Overall	≤40	41–69	≥70	
	N = 3451	N = 371	N = 2324	N = 756	*p*
Age at diagnosis, Median [IQR]	58.0 [47.0;68.0]	37.0 [33.0;39.0]	55.0 [48.0;62.0]	77.0 [73.0;81.0]	<0.001
Subtypes, n (%):					<0.001
HR+/HER2−	2358 (68.3%)	221 (59.6%)	1605 (69.1%)	532 (70.4%)	
HER2+	459 (13.3%)	61 (16.4%)	321 (13.8%)	77 (10.2%)	
Triple-negative	403 (11.7%)	73 (19.7%)	244 (10.5%)	86 (11.4%)	
Unknown	231 (6.7%)	16 (4.3%)	154 (6.6%)	61 (8.1%)	
Stage, n (%):					<0.001
I	1111 (32.2%)	58 (15.6%)	840 (36.1%)	213 (28.2%)	
II	1593 (46.2%)	215 (58.0%)	1030 (44.3%)	348 (46.0%)	
III	583 (16.9%)	83 (22.4%)	369 (15.9%)	131 (17.3%)	
IV	164 (4.8%)	15 (4.0%)	85 (3.7%)	64 (8.5%)	
Histology, n (%):					<0.001
Invasive ductal carcinoma	2765 (80.1%)	330 (88.9%)	1894 (81.5%)	541 (71.6%)	
Invasive lobular carcinoma	318 (9.2%)	8 (2.2%)	230 (9.9%)	80 (10.6%)	
Others	297 (8.6%)	29 (7.8%)	169 (7.3%)	99 (13.1%)	
Unknown	71 (2.1%)	4 (1.1%)	31 (1.3%)	36 (4.8%)	
Histologic grade, n (%):					<0.001
I	687 (19.9%)	45 (12.1%)	490 (21.1%)	152 (20.1%)	
II	1358 (39.4%)	120 (32.3%)	956 (41.1%)	282 (37.3%)	
III	965 (28.0%)	166 (44.7%)	600 (25.8%)	199 (26.3%)	
Not documented	441 (12.8%)	40 (10.8%)	278 (12.0%)	123 (16.3%)	
Ki67, n (%):					<0.001
<20%	1135 (32.9%)	61 (16.4%)	810 (34.9%)	264 (34.9%)	
≥20%	1669 (48.4%)	259 (69.8%)	1090 (46.9%)	320 (42.3%)	
Not documented	647 (18.7%)	51 (13.7%)	424 (18.2%)	172 (22.8%)	

**Table 2:** Treatment modalities and surgical approaches across age subgroups.

	Overall	≤40	41–69	≥70	
	N = 3451	N = 371	N = 2324	N = 756	*p*
First treatment, n (%):					<0.001
Surgery	2505 (72.6%)	200 (53.9%)	1760 (75.7%)	545 (72.1%)	
Neoadjuvant	792 (22.9%)	159 (42.9%)	487 (21.0%)	146 (19.3%)	
Palliative	124 (3.6%)	11 (3.0%)	64 (2.8%)	49 (6.5%)	
Others	30 (0.9%)	1 (0.3%)	13 (0.6%)	16 (2.1%)	
Type of surgery, n (%):					<0.001
Mastectomy	676 (20.5%)	146 (40.3%)	394 (17.4%)	136 (20.0%)	
Breast-conserving surgery	2138 (64.8%)	188 (51.9%)	1540 (68.2%)	410 (60.2%)	
Not documented	487 (14.8%)	28 (7.7%)	324 (14.3%)	135 (19.8%)	
Sentinel lymph node, n (%)	2165 (67.7%)	210 (60.2%)	1626 (74.3%)	329 (49.8%)	<0.001
Positive sentinel node, n (%)	728 (33.6%)	95 (45.2%)	537 (33.2%)	96 (28.2%)	<0.001
Type of sentinel node metastasis, n (%):					0.247
Micrometastasis (pN1mi)	340 (50.9%)	52 (59.1%)	243 (49.9%)	45 (48.4%)	
Macrometastasis (pN1a)	328 (49.1%)	36 (40.9%)	244 (50.1%)	48 (51.6%)	
Lymphadenectomy, n (%)	1310 (43.0%)	195 (59.5%)	861 (41.6%)	254 (39.2%)	<0.001
Adjuvant chemotherapy, n (%)	1233 (35.7%)	157 (42.3%)	966 (41.6%)	110 (14.6%)	<0.001
Trastuzumab, n (%) ^1^	446 (36.2%)	72 (45.9%)	322 (33.3%)	52 (47.2%)	<0.001
Status ^2^, n (%):					<0.001
Deceased	891 (25.8%)	61 (16.4%)	408 (17.6%)	422 (55.8%)	
Alive	2560 (74.2%)	310 (83.6%)	1916 (82.4%)	334 (44.2%)	
Follow-up time (years), Mean (SD)	9.9 (3.5)	10.5 (3.2)	10.5 (3.0)	8.0 (4.2)	

^1^ Percentages computed out of patients who received adjuvant therapy; ^2^ Administrative censoring at 30 November 2023.

**Table 3:** Five- and ten-year OS according to prognostic factors and age subgroups.

	Total		≤40		41–69		≥70	
	5-Year	10-Year	5-Year	10-Year	5-Year	10-Year	5-Year	10-Year
Global	86% (85%, 87%)	77% (76%, 78%)	89% (86%, 92%)	85% (81%, 88%)	91% (90%, 92%)	85% (83%, 86%)	70% (66%, 73%)	50% (47%, 54%)
Histologic grade								
I	94 (93, 96)	86 (84, 89)	100 (100, 100)	96 (90, 100)	97 (95, 98)	93 (91, 95)	86 (80, 91)	63 (56, 71)
II	90 (88, 91)	81 (79, 83)	96 (92, 99)	94 (90, 98)	93 (92, 95)	86 (84, 88)	74 (69, 79)	57 (51, 63)
III	80 (78, 83)	71 (68, 74)	83 (77, 89)	78 (72, 85)	87 (84, 89)	79 (76, 82)	60 (53, 67)	40 (34, 48)
Not documented	76 (72, 80)	65 (61, 70)	85 (75, 97)	70 (57, 86)	84 (80, 88)	77 (72, 82)	56 (48, 66)	37 (30, 47)
Subtypes								
HR+/HER2−	89 (88, 91)	80 (78, 81)	95 (92, 98)	90 (86, 94)	93 (92, 95)	87 (85, 88)	75 (72, 79)	55 (51, 59)
HER2+	86 (83, 90)	78 (74, 82)	92 (85, 99)	87 (79, 96)	92 (89, 95)	85 (81, 89)	61 (51, 73)	42 (32, 54)
Triple-negative	70 (66, 75)	62 (58, 67)	70 (60, 81)	67 (57, 79)	78 (73, 83)	71 (65, 77)	49 (39, 61)	35 (26, 47)
Not classifiable	81 (76, 86)	73 (68, 79)	88 (73, 100)	81 (64, 100)	88 (83, 94)	82 (77, 89)	59 (48, 73)	47 (36, 62)
Stage								
I	94 (93, 96)	88 (86, 90)	98 (95, 100)	97 (92, 100)	97 (96, 98)	92 (91, 94)	83 (78, 88)	66 (60, 73)
II	89 (88, 91)	80 (78, 82)	93 (90, 97)	90 (86, 94)	93 (92, 95)	87 (85, 89)	75 (71, 80)	54 (49, 59)
III	79 (76, 83)	66 (62, 70)	80 (71, 89)	70 (61, 80)	86 (83, 90)	75 (71, 80)	60 (52, 69)	37 (30, 47)
IV	25 (19, 33)	16 (11, 23)	47 (27, 80)	40 (22, 74)	31 (22, 42)	17 (11, 28)	12 (6.5, 24)	7.8 (3.4, 18)

Administrative censoring at 30 November 2023.

**Table 4:** Multivariate Cox Model for risk factors associated with mortality.

	Hazard Ratio	95% CI	*p*
**Age**			
41–69	1		
≤40	0.29	0.07–1.25	0.0968
≥70	4.90	3.44–6.97	<0.001
**Subtypes**			
HR+/HER2−	1		
HER2+	0.91	0.66–1.25	0.5591
Triple negative	1.63	1.20–2.22	0.0018
**Stage**			
I	1		
II	1.57	1.17–2.11	0.0030
III	2.84	2.04–3.96	<0.001
IV	20.9	14.2–30.7	<0.001
**Histology**			
I	1		
II	1.32	1.05–1.65	0.0156
III	1.80	1.41–2.29	<0.001
**Age** × **subtype**			
≤40 × HR+/HER2−	1		
≤40 × HER2+	0.98	0.37–2.57	0.9680
≥70 × HER2+	1.17	0.73–1.87	0.5038
≤40 × Triple-negative	2.25	1.11–4.57	0.0240
≥70 × Triple-negative	0.89	0.59–1.35	0.5847
**Age** × **Stage**			
≤40 × I	1		
≤40 × II	1.26	0.28–5.61	0.7651
≥70 × II	0.95	0.63–1.44	0.8100
≤40 × III	2.85	0.64–12.7	0.1690
≥70 × III	0.69	0.43–1.10	0.1211
≤40 × IV	1.92	0.37–10.1	0.4417
≤70 × IV	0.36	0.20–0.63	<0.001

## Data Availability

The datasets used and/or analyzed during the current study are available from the corresponding author on reasonable request.

## Supplementary Materials

The following supporting information can be downloaded at: https://www.mdpi.com/article/10.3390/cancers17081366/s1, Figure S1: Flowchart; Figure S2: Probability of survival divided by groups of age and subtype, histologic grade and stage; Figure S3: Probability of survival in HR+/HER2− according to tumor histological grade low versus high; Figure S4: Relative survival by group of age at diagnosis; Table S1: Description of the variables in the study; Table S2: Demographic data; data distribution by tumor subtype; Table S3: Demographic data; data distribution by clinical stage; Table S4: Median OS by characteristics and prognostic factors across age subgroups; Table S5: Five- and ten-year OS by stages and subtypes.

## Abbreviations

The following abbreviations are used in this manuscript:

ICO	Institut Català d’Oncologia
BC	Breast Cancer
BCS	Breast-Conserving Surgery
TNBC	Triple-Negative Breast Cancer
HR+	Hormone Receptor-Positive
HR	Hazard Ratio
OS	Overall Survival
GROW	Guidance for Reporting Oncology Real-World Evidence
DB	Database
IQR	Interquartile Range
CI	Confidence Interval

## References

[1] Sisti A., Huayllani M.T., Boczar D., Restrepo D.J., Spaulding A.C., Emmanuel G., Bagaria S.P., McLaughlin S.A., Parker A.S., Forte A.J. (2020). Breast cancer in women: A descriptive analysis of the national cancer database. Cancers.

[2] Wu X., Hildebrandt M.A., Ye Y., Chow W.-H., Gu J., Cunningham S., Zhao H., Hawk E.T., Wagar E., Rodriguez A. (2016). Cohort profile: The MD Anderson cancer patients and survivors cohort (MDA-CPSC). Int. J. Epidemiol..

[3] Tada K., Kumamaru H., Miyata H., Asaga S., Iijima K., Ogo E., Kadoya T., Kubo M., Kojima Y., Tanakura K. (2023). Characteristics of female breast cancer in japan: Annual report of the National Clinical Database in 2018. Breast Cancer.

[4] Freedman R.A., Partridge A.H. (2017). Emerging data and current challenges for young, old, obese, or male patients with breast cancer. Clin. Cancer Res..

[5] Walbaum B., García-Fructuoso I., Martínez-Sáez O., Schettini F., Sánchez C., Acevedo F., Chic N., Muñoz-Carrillo J., Adamo B., Muñoz M. (2024). Hormone receptor-positive early breast cancer in young women: A comprehensive review. Cancer Treat. Rev..

[6] Partridge A.H., Hughes M.E., Warner E.T., Ottesen R.A., Wong Y.-N., Edge S.B., Theriault R.L., Blayney D.W., Niland J.C., Winer E.P. (2016). Subtype-Dependent Relationship Between Young Age at Diagnosis and Breast Cancer Survival. J. Clin. Oncol..

[7] Azim H.A., Partridge A.H. (2014). Biology of breast cancer in young women. Breast Cancer Res..

[8] Thomas A., Rhoads A., Pinkerton E., Schroeder M.C., Conway K.M., Hundley W.G., McNally L.R., Oleson J., Lynch C.F., Romitti P.A. (2019). Incidence and Survival Among Young Women With Stage I-III Breast Cancer: SEER 2000–2015. JNCI Cancer Spectrum.

[9] Liu Z., Sahli Z., Wang Y., Wolff A.C., Cope L.M., Umbricht C.B. (2018). Young age at diagnosis is associated with worse prognosis in the Luminal A breast cancer subtype: A retrospective institutional cohort study. Breast Cancer Res. Treat..

[10] Vuong B., Jacinto A.I., Chang S.B., Kuehner G.E., Savitz A.C. (2024). Contemporary Review of the Management and Treatment of Young Breast Cancer Patients. Clin. Breast Cancer.

[11] Lee S.Y., Seo J.H. (2018). Current Strategies of Endocrine Therapy in Elderly Patients with Breast Cancer. Biomed. Res. Int..

[12] Rassu P.C. (2021). Breast surgical oncology in elderly and unfit patients: A systematic review. Minerva Surg..

[13] Fadda G.M., Santeufemia D.A., Basso S.M., Tozzoli R., Falcomer F., Lumachi F. (2016). Adjuvant Treatment of Early Breast Cancer in the Elderly. Med. Chem..

[14] Castelo-Branco L., Pellat A., Martins-Branco D., Valachis A., Derksen J.W.G., Suijkerbuijk K.P.M., Dafni U., Dellaporta T., Vogel A., Prelaj A. (2023). ESMO Guidance for Reporting Oncology real-World evidence (GROW). Ann. Oncol..

[15] Human Mortality Database. https://www.mortality.org/.

[16] Paluch-Shimon S., Cardoso F., Partridge A., Abulkhair O., Azim H., Bianchi-Micheli G., Cardoso M., Curigliano G., Gelmon K., Gentilini O. (2022). ESOeESMO fifth international consensus guidelines for breast cancer in young women (BCY5). Ann. Oncol..

[17] Culha Y., Davarci S.E., Ünlü B., Özaşkin D., Demir H., Baykara M. (2024). Comparison of clinicopathological and prognostic features of breast cancer patients younger than 40 years and older than 65 years. Discov. Oncol..

[18] Liedtke C., Hess K.R., Karn T., Rody A., Kiesel L., Hortobagyi G.N., Pusztai L., Gonzalez-Angulo A.M. (2013). The prognostic impact of age in patients with triple-negative breast cancer. Breast Cancer Res. Treat..

[19] Rottenberg Y., Naeim A., Uziely B., Peretz T., Jacobs J.M. (2018). Breast cancer among older women: The influence of age and cancer stage on survival. Arch. Gerontol. Geriatr..

[20] Fietz T., Zahn M.-O., Köhler A., Engel E., Frank M., Kruggel L., Jänicke M., Marschner N., The TMK-Group (Tumour Registry Breast Cancer) (2018). Routine treatment and outcome of breast cancer in younger versus elderly patients: Results from the SENORA project of the prospective German TMK cohort study. Breast Cancer Res. Treat..

[21] Mohile S.G., Dale W., Somerfield M.R., Schonberg M.A., Boyd C.M., Burhenn P.S., Canin B., Cohen H.J., Holmes H.M., Hopkins J.O. (2018). Practical Assessment and Management of Vulnerabilities in Older Patients Receiving Chemotherapy: ASCO Guideline for Geriatric Oncology. J. Clin. Oncol..

[22] Königsberg R., Pfeiler G., Hammerschmid N., Holub O., Glössmann K., Larcher-Senn J., Dittrich C. (2016). Breast Cancer Subtypes in Patients Aged 70 Years and Older. Cancer Investig..

[23] Barthélémy P., Heitz D., Mathelin C., Polesi H., Asmane I., Litique V., Rob L., Bergerat J.-P., Kurtz J.-E. (2011). Adjuvant chemotherapy in elderly patients with early breast cancer. Impact of age and comprehensive geriatric assessment on tumor board proposals. Crit. Rev. Oncol. Hematol..

[24] Taira N., Sawaki M., Takahashi M., Shimozuma K., Ohashi Y. (2010). Comprehensive geriatric assessment in elderly breast cancer patients. Breast Cancer.

[25] Abdel-Razeq H., Rous F.A., Abuhijla F., Abdel-Razeq N., Edaily S. (2022). Breast Cancer in Geriatric Patients: Current Landscape and Future Prospects. Clin. Interv. Aging.

[26] Torregrosa-Maicas M.D., del Barco-Berrón S., Cotes-Sanchís A., Lema-Roso L., Servitja-Tormo S., Gironés-Sarrió R. (2022). Expert consensus to optimize the treatment of elderly patients with luminal metastatic breast cancer. Clin. Transl. Oncol..

[27] Dotan E., Walter L.C., Beechinor R., Browner I.S., Carozza D., Clifton K., Cohen H.J., Dale W., Giri S., Gross C.P. (2023). NCCN Guidelines Version 1.2025. Older Adult Oncology NCCN Guidelines Panel Disclosures Continue NCCN. https://www.nccn.org.

[28] Wang S., Yang T., Qiang W., Shen A., Zhao Z., Yang H., Liu X. (2022). The prevalence of frailty among breast cancer patients: A systematic review and meta-analysis. Support. Care Cancer.

[29] Schmidt M., Loibl S. (2024). Chemotherapy in older patients with early breast cancer. Breast.

[30] De Glas N., Bastiaannet E., De Boer A., Siesling S., Liefers G.J., Portielje J. (2019). Improved survival of older patients with advanced breast cancer due to an increase in systemic treatments: A population-based study. Breast Cancer Res. Treat..

