# Supplementary material for: Comprehensive Clinical Characterization and Long-Term Follow-Up of the Institut Català d’Oncologia Breast Cancer Observational Cohort Study
Source: Cancers (Basel). 2025 Apr 19;17(8):1366. doi: 10.3390/cancers17081366 (PMC12025695; doi:10.3390/cancers17081366)
Supplement: Supplementary file 1 [file cancers-17-01366-s001.zip › cancers-3471806-supplementary.pdf]

Supplementary Materials

This supplementary file has been provided by the authors to give readers additional information about their work.

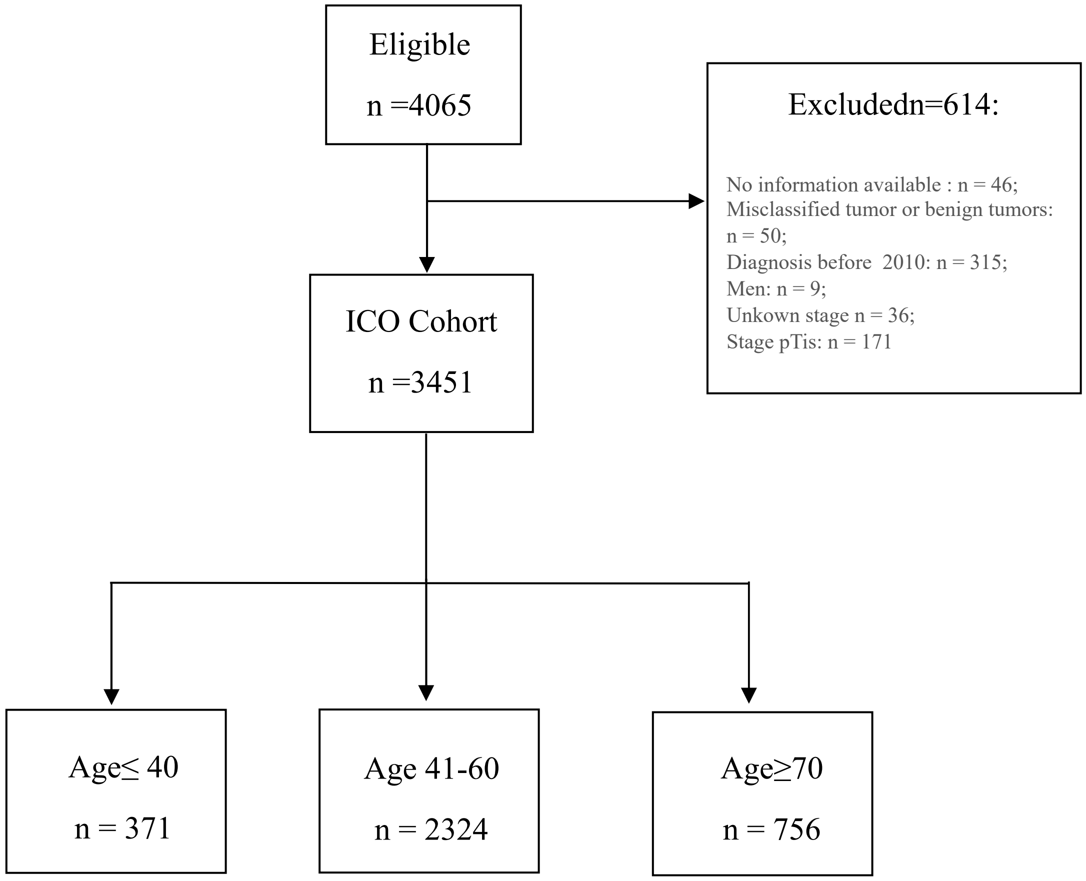

Figure S1. Flowchart.

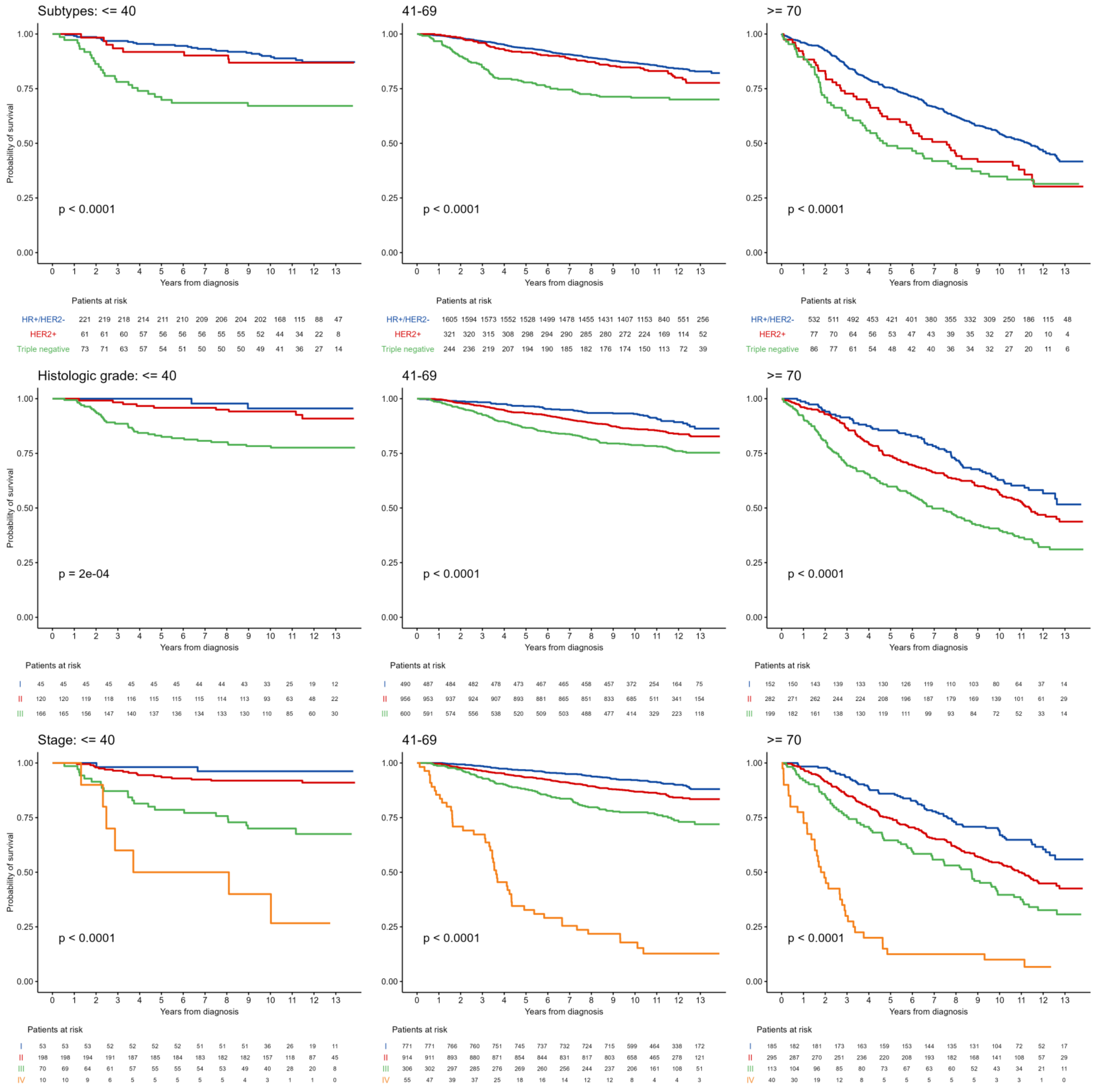

Figure S2. Probability of survival divided by groups of age and subtype, histologic grade and stage.

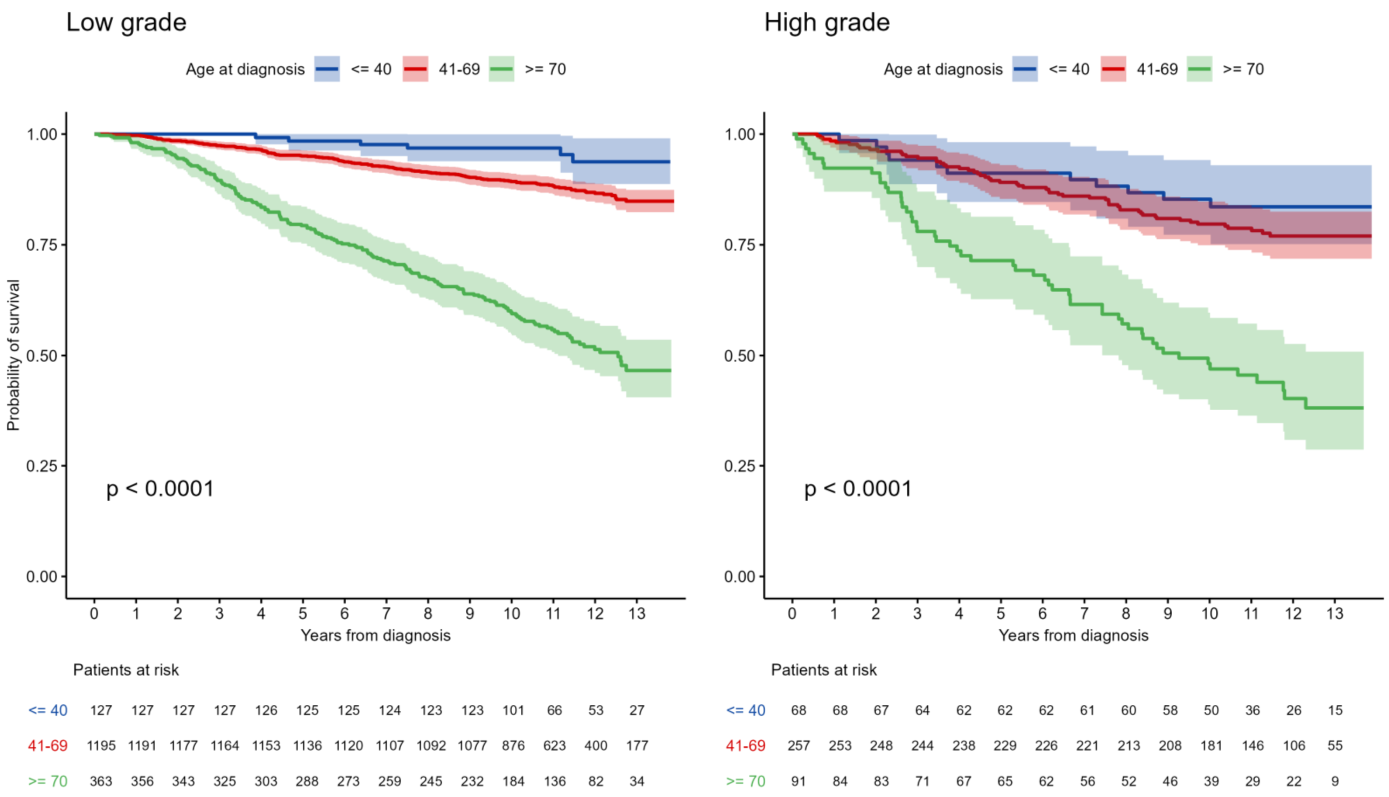

Figure S3. Probability of survival in HR+/HER2- according to tumor histological grade low versus high.

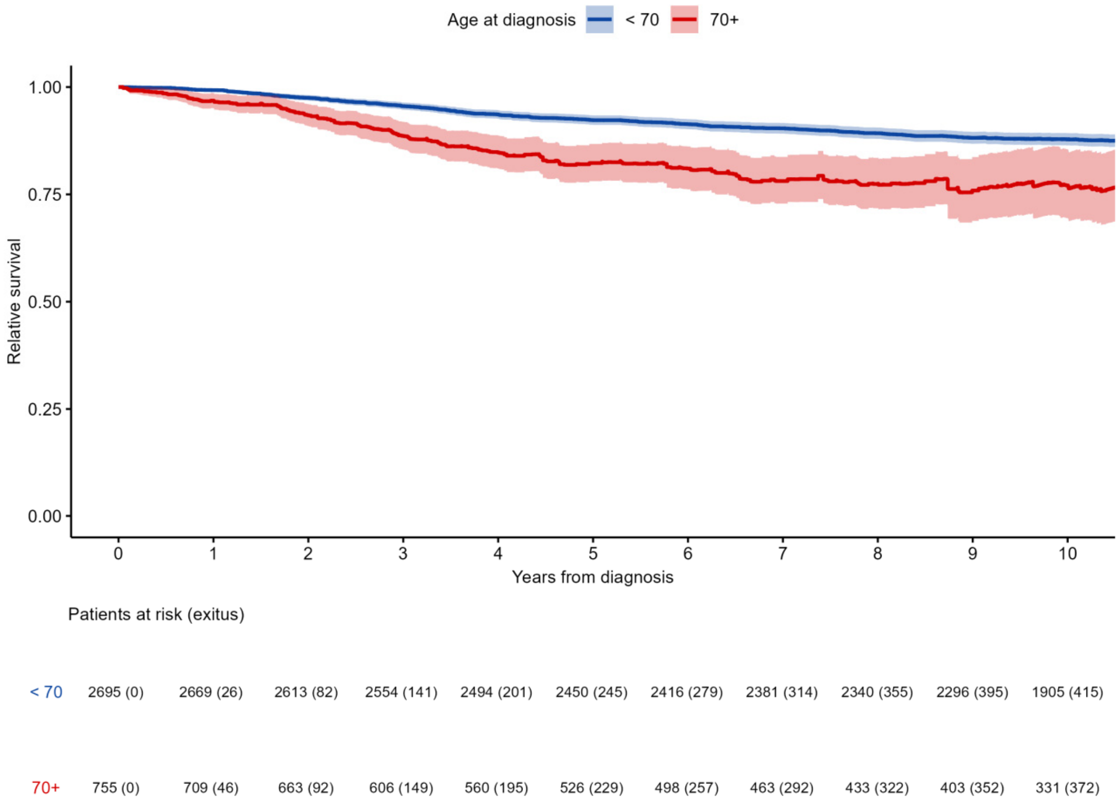

	**Overall survival (95% CI)**		**Relative survival (95% CI)**	
	**< 70**	**>=70**	**< 70**	**>=70**
**2-years**	97% (96%, 98%)	88% (85%, 90%)	97% (97%, 98%)	93% (91%, 96%)
**5-years**	91% (90%, 92%)	70% (66%, 73%)	92% (91%, 93%)	82% (78%, 87%)
**10-years**	85% (83%, 86%)	50% (47%, 54%)	88% (86%, 89%)	77% (69%, 86%)

Figure S4. Relative survival by group of age at diagnosis.

Table of contents

Table S1. Description of the variables in the study.

Groups of variables	Nº	Description of variables
Administrative	4	ICO Center (*Badalona, Girona, L'Hospitalet*)<br>Source of death<br>Source of last contact not ICO (ICO, Not ICO, ODR)<br>Official death registry acceptance level (ODR)
Demographics	3	Sex (biologic): woman/man<br>Date of birth<br>Age at diagnosis: calculated
Diagnostics & prognostic factors	21	Tumor type (generic = breast cancer)<br>Clinical T<br>Clinical N<br>M<br>Pathological T<br>Pathological N<br>Initial stage (specific breast eighth ed. TNM)<br>ECOG at diagnosis (0, 1, 2, 3, 4)<br>First biopsy pathology result<br>First biopsy date<br>Final pathology result (surgical specimen)<br>Final pathology date<br>Histological grade I, II, III)<br>Ki67 (%)<br>EERR (%)<br>PPRR (%)<br>HER2: + / ++/ +++<br>If HER2 ++: FISH positive, FISH negative<br>Positive sentinel lymph node (SLN)<br>If SLN positive: Micro/Macro metastasis<br>Negative sentinel lymph node
Surgery	8	Perform surgery: yes/no<br>Type of surgery: lumpectomy, quadrantectomy, hemi mastectomy, simple mastectomy, radical mastectomy, surgical biopsy<br>Date of surgery<br>Sentinel lymph node: Yes/no<br>Axillary lymphadenectomy: Yes/no<br>Conservative surgery: yes/no<br>Mastectomy reconstruction yes/no<br>If reconstruction: Immediate/Delayed
Systemic treatments	16	Perform chemotherapy (CT): yes/no<br>CT modality: adjuvant, neoadjuvant, concomitant, palliative<br>CT protocol type<br>Other type of CT<br>CT start date<br>CT end date<br>Targeted therapy (TT): yes/no<br>Type of TT: Trastuzumab

		Start date of TT<br>Hormone therapy (HT): yes/no<br>HT modality: adjuvant, neoadjuvant, concomitant, palliative<br>Type of HT<br>Start date of HT<br>End date of HT<br>Participates in trial yes/no<br>Trial start date
Radiotherapy	9	Performs external radiotherapy (RT): yes/no<br>RT location: local, local plus nodes, metastasis<br>RT modality: concomitant, sequential, exclusive, preoperative, postoperative without CT<br>Total RT dose<br>RT start date<br>RT end date<br>Performs Brachytherapy (BT): yes/no<br>BT start date<br>BT end date
Evolution and monitoring	12	Response to treatment (only initial protocol)<br>Anatomy-pathological response to neoadjuvant treatment<br>Relapse: yes/no (only if Complete Response has been done previously)<br>Date of relapse<br>Type of relapse<br>Progression: yes/no<br>Date of progression<br>Type of progression<br>Patient status at last check-up<br>Date of last check-up in ICO<br>Date of last check-up not in ICO<br>Date of last check-up of Official death registry (ODR): only in not dead
Dead	4	Dead: yes/no<br>Date of death (recorded in electronic register)<br>ODR date<br>Cause of death

Note: ICO: "*InstitutCatalàd'Oncologia*"; ODR: Official death registry; TNM for specific breast eighth edition; SLN: sentinel lymph node; EERR: estrogen receptors; PPRR: progesterone receptors; CT: chemotherapy; TT: targeted therapy; HT: hormone therapy; RT: radiotherapy; BT: brachytherapy.

Table S2. Demographic data; data distribution by tumor subtype.

	**Overall**	**HR+/HER2-**	**HER2+**	**Triple negative**	**Notclassifiable**
	**N=3451**	**N=2358**	**N=459**	**N=403**	**N=231**
**Ageat diagnosis, Mean [range]**	58.0 [19.0;98.0]	58.0 [19.0;98.0]	54.0 [22.0;95.0]	56.0 [23.0;93.0]	58.0 [27.0;97.0]
**≤ 40**	371 (10.8%)	221 (9.4%)	61 (13.3%)	73 (18.1%)	16 (6.9%)
**41-69**	2324 (67.3%)	1605 (68.1%)	321 (69.9%)	244 (60.5%)	154 (66.7%)
**≥ 70**	756 (21.9%)	532 (22.6%)	77 (16.8%)	86 (21.3%)	61 (26.4%)
**Stage, n (%):**					

**I**	1111 (32.2%)	814 (34.5%)	110 (24.0%)	90 (22.3%)	97 (42.0%)
**II**	1593 (46.2%)	1086 (46.1%)	225 (49.0%)	204 (50.6%)	78 (33.8%)
**III**	583 (16.9%)	371 (15.7%)	91 (19.8%)	85 (21.1%)	36 (15.6%)
**IV**	164 (4.8%)	87 (3.7%)	33 (7.2%)	24 (6.0%)	20 (8.7%)
**Histology, n (%):**					
**Invasive ductal carcinoma**	2765 (80.1%)	1878 (79.6%)	393 (85.6%)	322 (79.9%)	172 (74.5%)
**Invasive lobular carcinoma**	318 (9.2%)	280 (11.9%)	19 (4.1%)	7 (1.7%)	12 (5.2%)
**Others**	297 (8.6%)	167 (7.1%)	33 (7.2%)	62 (15.4%)	35 (15.2%)
**Unknown**	71 (2.1%)	33 (1.4%)	14 (3.1%)	12 (3.0%)	12 (5.2%)
**Histologic grade, n (%):**					
**I**	687 (19.9%)	611 (25.9%)	18 (3.9%)	14 (3.5%)	44 (19.0%)
**II**	1358 (39.4%)	1074 (45.5%)	154 (33.6%)	59 (14.6%)	71 (30.7%)
**III**	965 (28.0%)	416 (17.6%)	221 (48.1%)	285 (70.7%)	43 (18.6%)
**Not documented**	441 (12.8%)	257 (10.9%)	66 (14.4%)	45 (11.2%)	73 (31.6%)
**Ki67, n (%):**					
**<20%**	1135 (32.9%)	1009 (42.8%)	59 (12.9%)	22 (5.5%)	45 (19.5%)
**≥20%**	1669 (48.4%)	988 (41.9%)	320 (69.7%)	303 (75.2%)	58 (25.1%)
**Not documented**	647 (18.7%)	361 (15.3%)	80 (17.4%)	78 (19.4%)	128 (55.4%)
**First treatment, n (%):**					
**Surgery**	2505 (72.6%)	1870 (79.3%)	249 (54.2%)	209 (51.9%)	177 (76.6%)
**Neoadjuvant**	792 (22.9%)	407 (17.3%)	183 (39.9%)	170 (42.2%)	32 (13.9%)
**Palliative**	124 (3.6%)	69 (2.9%)	22 (4.8%)	18 (4.5%)	15 (6.5%)
**Others**	30 (0.9%)	12 (0.5%)	5 (1.1%)	6 (1.5%)	7 (3.0%)
**Type of surgery, n (%):**					
**Mastectomy**	676 (20.5%)	443 (19.5%)	130 (29.6%)	73 (19.2%)	30 (14.6%)
**Breast-conserving surgery**	2138 (64.8%)	1515 (66.6%)	249 (56.7%)	249 (65.5%)	125 (60.7%)
**No documented**	487 (14.8%)	318 (14.0%)	60 (13.7%)	58 (15.3%)	51 (24.8%)
**Sentinel node, n (%)**	2165 (67.7%)	1554 (70.4%)	246 (58.2%)	222 (60.2%)	143 (72.6%)
**Positive sentinel node, n (%)**	728 (33.6%)	557 (35.7%)	83 (34.0%)	47 (21.2%)	41 (29.1%)
**Type of sentinel node metastasis, n (%):**					

**Micrometastasis (pN1mi)**	340 (50.9%)	253 (48.9%)	42 (58.3%)	23 (56.1%)	22 (57.9%)
**Macrometastasis (pN1a)**	328 (49.1%)	264 (51.1%)	30 (41.7%)	18 (43.9%)	16 (42.1%)
**Lymphadenectomy, n (%)**	1310 (43.0%)	834 (39.7%)	231 (56.8%)	174 (49.3%)	71 (37.8%)
**Status1, n (%):**					
**Deceased**	891 (25.8%)	547 (23.2%)	119 (25.9%)	154 (38.2%)	71 (30.7%)
**Alive**	2560 (74.2%)	1811 (76.8%)	340 (74.1%)	249 (61.8%)	160 (69.3%)
**Time on follow-up (years), Mean (SD)**	9.9 (3.5)	10.2 (3.1)	10.0 (3.4)	8.5 (4.5)	9.8 (4.1)

1: AdministrativecensoringatNovember 30 2023

Table S3. Demographic data; data distribution by clinical stage.

	**Total**	**I**	**II**	**III**	**IV**
	**N=3451**	**N=1111**	**N=1593**	**N=583**	**N=164**
**Age at diagnosis, Mean [range]**	58.0 [19.0;98.0]	59.0 [27.0;93.0]	56.0 [19.0;97.0]	56.0 [25.0;96.0]	63.0 [32.0;98.0]
**≤ 40**	371 (10.8%)	58 (5.2%)	215 (13.5%)	83 (14.2%)	15 (9.1%)
**41-69**	2324 (67.3%)	840 (75.6%)	1030 (64.7%)	369 (63.3%)	85 (51.8%)
**≤70**	756 (21.9%)	213 (19.2%)	348 (21.8%)	131 (22.5%)	64 (39.0%)
**Subtype, n (%):**					
**HR+/HER2-**	2358 (68.3%)	814 (73.3%)	1086 (68.2%)	371 (63.6%)	87 (53.0%)
**HER2+**	459 (13.3%)	110 (9.9%)	225 (14.1%)	91 (15.6%)	33 (20.1%)
**Triple negative**	403 (11.7%)	90 (8.1%)	204 (12.8%)	85 (14.6%)	24 (14.6%)
**Not classifiable**	231 (6.7%)	97 (8.7%)	78 (4.9%)	36 (6.2%)	20 (12.2%)
**Histology, n (%):**					
**Invasive ductal carcinoma**	2765 (80.1%)	957 (86.1%)	1284 (80.6%)	448 (76.8%)	76 (46.3%)
**Invasive lobular carcinoma**	318 (9.2%)	68 (6.1%)	154 (9.7%)	83 (14.2%)	13 (7.9%)
**Others**	297 (8.6%)	83 (7.5%)	148 (9.3%)	49 (8.4%)	17 (10.4%)
**Unknown**	71 (2.1%)	3 (0.3%)	7 (0.4%)	3 (0.5%)	58 (35.4%)
**Histologic grade, n (%):**					
**I**	687 (19.9%)	327 (29.4%)	284 (17.8%)	66 (11.3%)	10 (6.1%)
**II**	1358 (39.4%)	460 (41.4%)	643 (40.4%)	209 (35.8%)	46 (28.0%)
**III**	965 (28.0%)	222 (20.0%)	480 (30.1%)	214 (36.7%)	49 (29.9%)
**Not documented**	441 (12.8%)	102 (9.2%)	186 (11.7%)	94 (16.1%)	59 (36.0%)
**Ki67, n (%):**					
**<20%**	1135 (32.9%)	486 (43.7%)	498 (31.3%)	117 (20.1%)	34 (20.7%)
**≥20%**	1669 (48.4%)	411 (37.0%)	824 (51.7%)	345 (59.2%)	89 (54.3%)

**Not documented**	647 (18.7%)	214 (19.3%)	271 (17.0%)	121 (20.8%)	41 (25.0%)
**First treatment, n (%):**					
**Surgery**	2505 (72.6%)	1088 (97.9%)	1123 (70.5%)	262 (44.9%)	32 (19.5%)
**Neoadjuvant**	792 (22.9%)	21 (1.9%)	459 (28.8%)	312 (53.5%)	0 (0.0%)
**Palliative**	124 (3.6%)	0 (0.0%)	0 (0.0%)	0 (0.0%)	124 (75.6%)
**Others**	30 (0.9%)	2 (0.2%)	11 (0.7%)	9 (1.5%)	8 (4.9%)
**Type of surgery, n (%):**					
**Mastectomy**	676 (20.5%)	68 (6.2%)	309 (19.6%)	268 (47.4%)	31 (57.4%)
**Breast-conserving surgery**	2138 (64.8%)	854 (77.4%)	1043 (66.1%)	224 (39.6%)	17 (31.5%)
**Not documented**	487 (14.8%)	181 (16.4%)	227 (14.4%)	73 (12.9%)	6 (11.1%)
**Sentinel node, n (%)**	2165 (67.7%)	975 (90.4%)	1055 (69.3%)	126 (23.9%)	9 (13.2%)
**Positive sentinel node, n (%)**	728 (33.6%)	23 (2.4%)	598 (56.9%)	101 (72.1%)	6 (54.5%)
**Type of sentinel node metastasis, n (%):**					
**Micrometastasis (pN1mi)**	340 (50.9%)	18 (78.3%)	304 (54.7%)	16 (18.8%)	2 (50.0%)
**Macrometastasis (pN1a)**	328 (49.1%)	5 (21.7%)	252 (45.3%)	69 (81.2%)	2 (50.0%)
**Lymphadenectomy, n (%)**	1310 (43.0%)	53 (5.4%)	701 (48.6%)	511 (93.2%)	45 (65.2%)
**Status[1], n (%):**					
**Deceased**	891 (25.8%)	167 (15.0%)	361 (22.7%)	220 (37.7%)	143 (87.2%)
**Alive**	2560 (74.2%)	944 (85.0%)	1232 (77.3%)	363 (62.3%)	21 (12.8%)
**Time on follow-up (years), Mean (SD)**	9.9 (3.5)	10.9 (2.6)	10.2 (3.1)	9.2 (3.9)	4.0 (3.8)
1: AdministrativecensoringatNovember 30 2023					

Table S4. Median OS by characteristics and prognostic factors across age subgroups.

	**Total**	**≤ 40**	**41-69**	**≥ 70**
	**Median OS (95% CI)**	**Median OS (95% CI)**	**Median OS (95% CI)**	**Median OS (95% CI)**
**Global**	NR	NR	NR	10 (8.9, 11)
**Histologic grade**				
**I**	NR	NR	NR	NR (12, NR)
**II**	NR	NR	NR	11 (10, NR)
**III**	NR	NR	NR	6.9 (5.9, 8.9)
**Not documented**	NR	NR	NR	6.5 (4.6, 8.5)
**Subtypes**				
**HR+/HER2-**	NR	NR	NR	11 (10, 12)
**HER2+**	NR	NR	NR	7.6 (5.4, 11)
**Triple negative**	NR	NR	NR	4.7 (3.6, 8.7)
**Not classifiable**	NR	NR	NR	8.7 (4.5, NR)

Stage				
I	NR	NR	NR	NR (12, NR)
II	NR	NR	NR	11 (9.5, 12)
III	NR	NR	NR	7.5 (5.8, 9.6)
IV	2.9 (2.3, 3.3)	3.7 (2.3, NR)	3.5 (3.1, 4.2)	2.0 (1.6, 2.7)
**NR: Not reached**				

Table S5. Five- and ten-years OS by stages and subtypes.

	**HR+/HER2-**			**HER2+**			**TNBC**	
	**5-years**	**10-years**		**5-years**	**10-years**		**5-years**	**10-years**
**Stage**								
**I**	95% (93%, 96%)	89% (86%, 91%)		95% (92%, 99%)	88% (82%, 94%)		92% (87%, 98%)	83% (76%, 91%)
**II**	92% (91%, 94%)	82% (80%, 84%)		92% (88%, 95%)	84% (79%, 89%)		73% (67%, 79%)	66% (59%, 73%)
**III**	85% (81%, 88%)	70% (65%, 74%)		79% (71%, 88%)	68% (59%, 78%)		60% (50%, 71%)	49% (40%, 61%)
**IV**	28% (20%, 39%)	16% (9.8%, 26%)		42% (29%, 63%)	30% (18%, 51%)		4.2% (0.6%, 28%)	4.2% (0.6%, 28%)

ICO-Breast Cancer Cohort Working group (project and database design, data collection and statistical analysis):

José Ramón Germà (medical oncologist, project manager, ICO Hospitalet); Pablo López-Garcia (engineer database designer, ICO Hospitalet); Lourdes Pétriz (radiation oncologist, project coordinator, ICO Hospitalet); Joan Muniesa (informatics systems, ICO Hospitalet); Eva Loureiro (informatics systems, ICO Hospitalet); Anna Esteve (statistical analysis, ICO-Badalona); Paula Rodriguez-Blanco (data entry, ICO Hospitalet); Francisca Galdón (data entry, ICO Hospitalet); Roser Briculle (data entry, ICO Hospitalet); Ulises Ferrandiz (data entry, ICO Badalona); Gabriel Andrés (data entry, ICO Hospitalet)
